# Internalized and Perceived Stigma and Depression in Pulmonary Tuberculosis: Do They Explain the Relationship Between Drug Sensitivity Status and Adherence?

**DOI:** 10.3389/fpsyt.2022.869647

**Published:** 2022-05-19

**Authors:** Anmol Pradhan, Prakash Koirala, Samrat Singh Bhandari, Sanjiba Dutta, Pau García-Grau, Harshavardhan Sampath, Indralal Sharma

**Affiliations:** ^1^Department of Psychiatry, Sikkim Manipal Institute of Medical Sciences, Sikkim Manipal University, Gangtok, India; ^2^Department of Respiratory Medicine, Sikkim Manipal Institute of Medical Sciences, Sikkim Manipal University, Gangtok, India; ^3^Programa de Maestro de Educación Infantil, Universidad Católica de Valencia, Valencia, Spain

**Keywords:** pulmonary tuberculosis, multidrug-resistant tuberculosis (MDR-TB), internalized stigma, depression, prevalence, Patient Health Questionnaire-9, medication adherence

## Abstract

**Background:**

Adherence to medication for tuberculosis (TB) has been found to be deleteriously affected by psychosocial issues, including internalized and perceived stigma (IPS) and depression, usually resulting in the emergence of multidrug-resistant TB (MDR-TB). The objective of the study was to find the prevalence of depression among patients receiving treatment for pulmonary TB, and how stigma and depression affect the relationship between drug sensitivity status (DSS) and treatment adherence.

**Method:**

It was a cross-sectional observational study conducted between January 2019 and July 2020 in two centers in Sikkim, India. The Patient Health Questionnaire-9 (PHQ-9), Internalized Social Stigma Scale (ISSS), and Tuberculosis Medication Adherence Scale were used to assess depression, IPS, and medication adherence, respectively. A path analysis was performed with DSS, treatment adherence, IPS, and depression. Education in years was included in the model as it was significantly correlated with IPS.

**Results:**

A total of 71 patients who were on drug-sensitive TB (DS-TB) regimen (*n* = 26) and MDR-TB regimen (*n* = 45) participated in the study. Notably, 56.3% (*n* = 40) of the participants were found to have depression. Among the depressed participants, 32.5% were on the DS-TB regimen and 67.5% were on the MDR-TB regimen. The path analysis indicated that IPS and depression were serially mediating the relationship between DSS and treatment adherence (β = −0.06, *p* < 0.05, 95% CI = −3.20, −0.02). Finally, years of education had an exogenous predictor role, not only directly affecting IPS (β = −0.38, *p* < 0.001, 95% CI = −0.99, −0.31) but also affecting treatment adherence through IPS and depression (β = 0.08, *p* = 0.02, 95% CI = 0.03, 0.47). This indicated that with more years of education, the IPS decreases, which decreases depression and ultimately leads to better adherence.

**Conclusion:**

We found an important relationship between different psychosocial factors which may affect treatment adherence. Patients who have higher IPS are more likely to develop depression which negatively affect adherence. Patients on the MDR-TB regimen have higher stigma. There is an urgent need to integrate mental health services with TB Control Programs.

## Introduction

Tuberculosis (TB) is a communicable disease that is a major source of morbidity and one of the leading causes of mortality around the world. TB was the biggest cause of death from a single infectious agent until the coronavirus (COVID-19) pandemic, ranking ahead of HIV/AIDS (1). In 2020, the World Health Organization (WHO) regions of South-East Asia had the highest number of TB cases (43%), followed by Africa (25%), the Western Pacific region (18%) with lower numbers in the Eastern Mediterranean (8.3%), the America (3.0%), and Europe (2.3%). The National Tuberculosis Program, India, which was launched in 1962, was revitalized and renamed as Revised National TB Control Program (RNTCP) in 1993 after the WHO declared TB as a global emergency. A directly observed treatment-short course (DOTS) was launched in 1997 and by the late part of 2005, the whole country was covered (2). There are more than 850,000 cases of TB in India which are either undetected, untreated, or treated in private setup with poor-quality drugs or incorrect regime that contribute to the rise in the incidence of multidrug-resistant TB (MDR-TB) and extensively drug-resistant TB (XDR-TB) (3). A number of factors influence the health-seeking behavior of the affected individuals which includes awareness about the illness, accessibility to healthcare facilities, educational level, stigma (4–7), and comorbid conditions, such as anxiety and depression (8).

One of the primary reasons for difficulty in early detection and lost to follow-up of patients with TB in India is stigma (9). The term stigma is used to describe a severely disparaging attribute. There is a difference between enacted and felt stigma. Enacted stigma relates to instances of discrimination based on social unacceptability or inferiority, whereas perceived stigma is based on a profound sense of inferiority and refers to an oppressive fear of enacted stigma (7). Perceived stigma, such as fear of losing social status, fear of getting isolated in the community, fear of losing job, fear of poor marriage prospect, and fear of becoming a subject of gossip, has identified few of the patient-related factors that could impair active case detection (9, 10). Reluctance to avail the treatment because of stigma or irregular or poor adherence may lead to MDR-TB and XDR-TB (11). However, factors, such as higher education and knowledge about TB, have been found to have a buffering effect on the perception of stigma (6).

Depression has been found to be present among patients with TB and has been related to poor adherence to the prescribed treatment (12). Duko et al. reported that the pooled estimated prevalence of depression of 45.55% among patients with TB; additionally, they found in the subgroup analysis that the pooled prevalence of depression among patients with MDR-TB was higher than in patients without MDR-TB (13). Redwood et al. in their study performed in Vietnam found that people with MDR-TB scored higher in depression and stigma score when compared to people with drug-sensitive TB (DS-TB) (14). Ruiz-Grosso et al. in their meta-analysis found a strong association between depressive symptoms and negative treatment outcome. In addition, they found an association between depressive symptoms and loss of follow-up and increased mortality among patients with TB (15). In a study performed in South Ethiopia, it was found that patients with comorbid HIV-infection, poor social support, perceived stigma, currently having substance use disorder or if female, were more likely to have depression and anxiety. The same study reported a prevalence of depression and anxiety at 43.4 and 41.5%, respectively (16). Depression has been found to mediate the relationship between stigma and medication adherence among patients with TB. The study was performed in China (17). To the best of our knowledge, the complex interplay among drug sensitivity status (DSS; i.e., DS-TB and MDR-TB), perceived stigma, depression, and adherence to TB treatment has never been explored especially in the Indian scenario where cultural beliefs and orthodox societal norms are still prevalent. We also used measures that are specific and validated for patients with TB to assess for stigma and medication adherence. The objective of this survey was to find the prevalence of depression among the patients receiving DOTS. As the cognitive behavioral model of psychological phenomenon suggests that behaviors, thoughts, and emotions mutually influence each other (18), we proposed the following hypothesis:

H1: Internalized and perceived stigma (IPS) will be higher in patients on the MDR-TB regimen when compared to patients on the DS-TB regimen, and it will mediate the relationship between DSS and depression.
H2: Depression will mediate the relationship between IPS and treatment adherence.
H3: The relationship between treatment regimen and treatment adherence will be serially mediated by IPS and depression.
H4: Years of education will predict stigma and have an indirect effect on depression.

## Materials and Methods

### Study Design and Setting

This cross-sectional exploratory survey was conducted in two centers providing the treatment for both newly diagnosed cases and drug-resistant cases, in the East district of Sikkim, a small northeastern part of India bordering Bhutan, Nepal, and Tibet. These two centers are Central Referral Hospital (CRH), Sikkim Manipal Institute of Medical Sciences, and Sir Thutob Namgyal Memorial Hospital (STNMH). The survey was performed during the period of January 2019 to July 2020.

### Participants

All the patients who were 18 years of age and older and had completed 3 months of treatment for pulmonary TB were included in the study. As there was a lack of validated scale for measuring related physical symptoms, patients with extrapulmonary TB were not included. Patients who had a prior history of psychiatric illness before the diagnosis of TB and those who were having comorbid conditions, namely, HIV and hepatitis were also excluded from the study. The sample size was based on the number of patients diagnosed with pulmonary TB (excluding extensively drug-resistant pulmonary TB) receiving medication from the DOTS centers. On checking the records, 30 patients were on medication from CRH and 41 patients from STNMH. All 71 patients gave consent for participation.

### Measures

#### Sociodemographic Proforma

A structured proforma was used to record sociodemographic and clinical data. The sociodemographic data included gender, age, marital status, type of family, number of dependent family members, education, occupation status, monthly family income, and distance of DOTS center from current place of residence. Clinical data included months under treatment, type of pulmonary TB (e.g., miliary, cavitary, and multilobar), RNTCP regimen and medical comorbidity, RNTCP regimen (DS-TB regimen or MDR-TB regimen), and medical comorbidity. The quality of data was assured by checking the medical records and radiographic evidence.

#### Depression

Depression was assessed with the Patient Health Questionnaire-9 (PHQ-9). The scale consists of nine items scored on a 4-point Likert-type scale (from 0 to 3). The total score ranges from 0 to 27. Scores are divided into minimal (0–4), mild (5–9), moderate (10–14), moderately severe (15–19), and severe (20–27). The internal reliability test scored Cronbach’s alpha as 0.89 and 0.86 in two different studies (19). PHQ-9 was used as it takes less time and is more preferred in out-patient setting and was suitable for our study. It has been found to be strongly correlated with the Beck Depression Inventory (20). The psychometric properties of PHQ-9 for assessing depression among the Indian population have been found to be comparable with the finding of the Western studies (21).

#### Internalized and Perceived Stigma

The IPS was measured with a ten-item Internalized Social Stigma Scale (ISSS). ISSS was inspired by the scale developed by Boyd et al. within the context of mental illness (22). This ten-item scale has been found to have a good internal consistency (Cronbach’s alpha = 0.7) when tested among patients with TB (23). It measures four subdimensions, namely, alienation (the subjective experience of being less than a full member of the society or having a spoiled identity), perceived discrimination (perceptions of the way of patients with TB that they currently tend to be treated by others), stereotypes endorsement (the degree to which people affected by TB agree with common stereotypes about people with TB), and social withdrawal (the tendency of people affected by TB to isolate themselves from the rest of the society). A higher score indicates higher IPS (23).

#### Adherence to Tuberculosis Medication

Tuberculosis medication adherence scale (TBMAS) was developed by taking into consideration the difficulties experienced while assessing general medication adherence, such as pharmacy record review or pill counts, which is not feasible in clinical practice. TBMAS incorporates predictors of adherence, such as patient’s behavior and patient–provider interaction. TBMAS has 30 items which are scored in a 5-point Likert-type scale. Scores ranging from 30 to 150 with higher scores show better medication adherence. It assesses the following factors: communication with healthcare providers, confidence in curing TB, access to healthcare, social support, lifestyle and habits, personal traits, mood disorders, coping style, and forgetfulness. A higher score indicates better adherence (24). The internal consistency of the scores produced by the TBMAS scale in the Indian participants of the current study showed acceptable values, indicating an acceptable reliability. In addition to Cronbach’s alpha, the McDonald’s omega statistic (ω) was calculated, as Cronbach’s alpha underestimates the true reliability unless the items are tau-equivalent (25). In our study, the reliability of the scores was ω = 0.764, indicating acceptable values, with an average interitem correlation of *r* = 0.20, indicating all dimensions working well together and measuring the same overall construct.

## Ethical Review

The study was approved by the Institutional Ethical Committee of Sikkim Manipal University (SMIMS/IEC/2018-87) and was conducted according to the Declaration of Helsinki. All patients provided written informed consent prior to the study participation.

## Statistical Analyses

The aim of this study was first explored using descriptive statistics. To display the prevalence of depression among the participants, a PHQ-9 score greater than 5 was taken as depressed (19); for other analyses, it was taken as a continuous variable. An exploratory partial correlation analysis was performed to find out the correlation among years of education, DSS (coded as 0 = DS-TB and 1 = MDR-TB), IPS (ISSI score), TBMAS, and PHQ-9 with sociodemographic variables (age and income) controlled. A p-value of <0.05 was considered significant for all the tests. Analysis was performed using SPSS version 20 (IBM SPSS Statistics for Windows, Version 20.0., IBM Corp, Armonk, NY, United States). A path analysis was used to evaluate the fit of the theoretical model to the data. Path analyses allow the assessment of direct and indirect effects of a set of variables on a specific target variable, in this case, treatment adherence. The strength of the effects is represented by path coefficient, i.e., standardized partial regression coefficients with a range from −1 to +1. The fit of the data to the specified model was investigated using the χ2 test (26) and root mean square error of approximation (RMSEA) (27). In addition, the amount of variance in all endogenous variables was calculated through regression analysis. Effect sizes for both direct and indirect effects were calculated through standardized coefficients, due to the different scaling of some of the observed variables. R and Jamovi version 2.2 (Jamovi Project, 2021) were used for both the direct and indirect effects of the predictor variables on patients’ treatment adherence and the role of intermediate variables in indirect effects as a mediator. A *post-hoc* calculation for multiple regression analysis was employed using G*Power 3 with a probability value of.05, a medium effect size, and a sample size of *N* = 71. The statistical power for the analysis, considering our *N*, was 1-β = 0.712, *F*_(4,66)_ = 2.51, *p* = 0.05, *f*^2^ = 0.15, with four predictors. For a large effect size, however, the statistical power increased to.98 with four predictors (1-β = 0.982, *F*_(4,66)_ = 2.51, *p* = 0.05, *f*^2^ = 0.35), which was considered sufficient to test the hypothesized model.

## Results

The mean age of the participants was 28.14 years (SD = 10.46). Among the participants, 56.3% (*n* = 40) were found to have depression. Among the patients who were found to have depression, 32.5% (*n* = 13) were on the DS-TB regimen and 67.5% (*n* = 27) were on the MDR-TB regimen. The mean duration of treatment for patients on the DS-TB regimen and MDR-TB regimen was 3.81 (SD = 1.09) months and 9.20 (SD = 6.58) months, respectively. We found that the duration of treatment did not have any significant relation to PHQ-9 score. The details of the participants’ characteristics are given in Table 1.

**TABLE 1:** Characteristic of the participants (*N* = 71).

Variable	No. of patients (n)	Percentage (%)
**Gender**		
Male	35	49.3
Female	36	50.7
**Marital status**		
Unmarried	40	56.3
Married	31	43.7
**Occupation**		
Employed	37	52.1
Unemployed	34	47.9
**Type of family**		
Nuclear	50	70.4
Extended	19	26.8
Joint	2	2.8
**Education**		
No formal education	4	5.6
Primary school	7	9.9
Secondary school	23	32.4
Senior secondary school	18	25.4
Graduate	17	23.9
Post-graduate	2	2.8
**Pulmonary TB Type**		
Miliary	3	4.2
Cavitary	26	36.6
Multilobar	42	59.2
**DSS**		
DS-TB regimen	26	36.6
MDR-TB regimen	45	63.4

*DSS, drug sensitivity status; DS-TB, drug sensitive regimen; MDR-TB, multidrug resistant.*

### Correlation Analysis

In order to test the magnitude and plausibility of our research question, we looked at correlations between variables (Table 2). TB medication adherence was negatively and significantly correlated with depression, IPS related to TB, and DSS. Stigma was negatively correlated with years of education.

**TABLE 2:** Correlations and descriptive study of study variables.

	Yrs. of education (1)	DSS (2)	ISSS (3)	PHQ-9 (4)	TBMAS (5)	Mean (SD)
1	−					10.38(4.10) (yrs.)
2	–0.100	−				
3	−0.420**	0.306*	−			28.17(6.94)
4	–0.043	0.277*	0.409**	−		7.15(5.32)
5	0.018	–0.167	−0.266**	−0.580**	–	113.62(12.9)

**Correlation is significant at p < 0.01 (two-sided). *Correlation is significant at p < 0.05 (two-sided). ISSS, internalized social stigma scale; PHQ-9, patient health questionnaire-9; TBMAS, TB medication adherence scale. DSS was dichotomized as 0 = DS-TB, 1 = MDR-TB.

### Path Analysis

**FIGURE 1:**
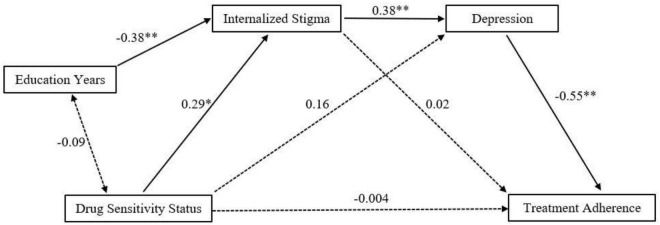
It shows the standardized coefficients for the paths of the model. Dashed arrows represent statistically non-significant paths, and solid arrows represent statistically significant paths.

All the four hypotheses (H1, H2, H3, and H4) were tested by path analysis. Overall, the model (Figure 1) was statistically significant (Akaike information criterion = 1,442; Bayesian information criterion = 1,471; χ^2^ = 2.63; *p* = 0.27), indicating that the data did not differ from the specified model (non-significant chi-square test). Additional fit measures indicated an optimum fit of the data to the model (RMSEA = 0.06 [0.00–0.25], *p* = 0.33); standardized root mean square residual = 0.04). Results from multiple regression analysis showed that the predictors had a statistically significant influence on all the endogenous variables, namely, for TBMAS, it was *R*^2^ = 0.29, *df* = 2, *p* < 0.001, for internal stigma, it was *R*^2^ = 0.25, *df* = 2, *p* < 0.001, and for depression, it was *R*^2^ = 0.20, *df* = 2, *p* < 0.001.

The results indicated that there was a statistically significant direct effect between IPS and depression, between education years and IPS, between DSS and IPS, and between depression and adherence to treatment (Table 3). We did not find any significant direct effect of DSS on depression. Even when IPS was included as a mediator, the indirect effect of DSS on depression remained insignificant (Figure 1). Three statistically significant indirect effects were found from DSS to TBMAS through IPS and depression (β = −0.06, *p* = 0.04). This result indicated that IPS and depression were serially mediating the relationship between DSS and treatment adherence. Whereas IPS was not directly related to TBMAS, there was a statistically significant indirect effect on TBMAS through depression (β = −0.20, *p* < 0.01). The negative beta indicated that the relationship was inverse and that the higher the IPS, the higher the depression and the lower the treatment adherence. Overall, IPS mediated the relationship of DSS and depression, depression mediated the relationship of IPS and treatment adherence, and both IPS and depression mediated the relationship between treatment regimen and treatment adherence.

**TABLE 3:** Direct and indirect path coefficients.

			95% CI				
Path	Estimate	SE	Lower	Upper	β	*z*	*p*
**Direct effect**							
PHQ9–>TBMAS	–1.338	0.273	−1.874	−0.802	−0.549	–4.894	<0.001
InStigma–>TBMAS	0.044	0.213	−0.373	0.461	0.024	0.207	0.836
DSS1–>TBMAS	–0.102	2.869	−5.725	5.521	−0.004	–0.035	0.972
DSS1–>PHQ9	1.804	1.227	−0.600	4.209	0.164	1.471	0.141
InStigma–>PHQ9	0.288	0.086	0.120	0.456	0.376	3.361	<0.001
DSS1–>InStigma	4.217	1.472	1.332	7.102	0.295	2.865	0.004
Edu_Yrs–>InStigma	–0.652	0.174	−0.994	−0.311	−0.385	–3.742	<0.001
**Indirect effect**							
InStigma ⇒ PHQ9 ⇒ TBMAS	–0.385	0.139	−0.658	−0.113	−0.206	–2.771	0.006
DSS1 ⇒ PHQ9 ⇒ TBMAS	–2.414	1.714	−5.774	0.945	−0.090	–1.409	0.159
DSS1 ⇒ InStigma ⇒ TBMAS	0.186	0.899	−1.576	1.947	0.007	0.206	0.836
DSS1 ⇒ InStigma ⇒ PHQ9 ⇒ TBMAS	–1.625	0.816	−3.225	−0.026	−0.061	–1.992	0.046
Edu_Yrs ⇒ InStigma ⇒ TBMAS	–0.029	0.139	−0.301	0.244	−0.009	–0.207	0.836
Edu_Yrs ⇒ InStigma ⇒ PHQ9 ⇒ TBMAS	0.251	0.113	0.030	0.473	0.079	2.227	0.026

*Estimate = Unstandardized structural coefficient, β = Standardized structural coefficient, SE = standard error of the structural coefficients. InStigma, internalized and perceived stigma; PHQ9, patient health questionnaire 9; DSS, drug sensitivity status was dichotomized as 0 = DS-TB, 1 = MDR-TB; TBMAS, TB medication adherence scale; Edu_Yrs, years of education.*

Finally, education years had an exogenous predictor role, not only directly affecting stigma (β = −0.38, *p* < 0.001) but also affecting treatment adherence through stigma and depression (β = 0.08, *p* = 0.02). This result indicated that with more years of education, the perceived and internal stigma decreases, which decreases depression and ultimately leads to better adherence.

## Discussion

In the current study, the prevalence of depression among patients with pulmonary TB receiving DOTS was 56.3%. More patients who were on the MDR-TB regimen reported depression when compared to patients on the DS-TB regimen. Although perceived and internal stigma and depression have been found to affect treatment adherence negatively, less is known about the possible complex interplay between different factors, such as sensitivity to drugs, education level of the affected individual, perceived stigma, depression, and how all these factors impact treatment adherence. The main aim of the study was to find whether DSS has any relationship with adherence to pulmonary TB and whether perceived stigma and depression together play a role in the influence of DSS on treatment adherence. We also wanted to find the role of completed years of education in this complex relationship. We found that stigma and depression sequentially mediated the relationship between DSS and adherence to treatment. We also found that patients with longer years of education had a lesser perceived or internal stigma.

Indian studies on the prevalence of depression among patients with TB reported findings from 16.1 to 80% (28, 29). We found that 56.3% of our patients had depression; such variation can be understood because of variation in the mean age of the participants, education level of population of the geographical region, social and cultural practice, as well as the screening instrument used. In their meta-analysis, Duko et al. reported a pooled prevalence of 45.19% (95% CI: 38.04–52.55), and they reported a high heterogeneity in the studies included in the analysis (16). These data may not be similar, and they do show a higher prevalence of depression among patients with TB when compared to the general population which is much lower, i.e., 5% (30). As one disease may lead to the development of the other, the interplay between TB and depression is complicated. Depression and stress can lead to the production of pro-inflammatory cytokines and also have been found to have an adverse effect on other immunological mechanisms, including the downregulation of cellular and humoral responses (31) which can contribute to the development of TB. In contrast, TB is a chronic inflammatory condition where there is the release in pro-inflammatory cytokines, such a like interleukin-β, tumor necrosis factor-α, and interferon-γ. These cytokines activate indoleamine-2,3-dioxygenase (IDO) which degrades tryptophan, a rate-limiting factor in serotonin synthesis resulting in depressive disorder. An increase in the activity of IDO results in the increasing production of quinolinic acid which is a neurotoxic metabolite of tryptophan and acts as an agonist for *N*-methyl-D-aspartate (NMDA) receptors. An increasing level of NMDA agonist again accelerates the development and worsening of depression (32).

Our research has some theoretical significance. The results of the path analysis showed that the DSS, whether on DS-TB or MDR-TB, did not predict depression by itself. However, when stigma was included as a mediator in the relationship, the finding indicates that the DSS affects depression through stigma. Patients who were on DOTS, both DS-TB and MDR-TB regimens, were likely to develop depression when the level of perceived and internal stigma increases beyond a certain level. Patients who were on the MDR-TB regimen have more perceived and internal stigma and depression when compared to those who were on the DS-TB regimen, and this is consistent with previous findings (13, 14, 33). In the same way, stigma did not have a direct effect on treatment adherence, but when depression was considered in the equation as a mediator, we found that a greater stigma predicted higher depression which resulted in lower adherence. Internal stigma due to TB may lead a person to self-imposed social isolation with a desire to protect their loved ones from getting infected. They may sleep alone, eat alone, and restrict their social communications with friends and families. This social isolation with uncertainty about treatment prognosis along with decreased functionality occupationally makes them lose their self-esteem and leads to the development of a sense of worthlessness, ultimately leading to clinical depression (12, 33) which may affect adherence by negatively affecting motivation and self-care. We also found that DSS did not predict treatment adherence by itself but the indirect effect was found through both perceived and internal stigma and depression. Finally, the path analysis showed that years of education could predict treatment adherence through the reduced perception of stigma and lower depression. Fear and ignorance that are considered to be the drivers of both perceived and actually experienced stigma can be reduced by education (34). Higher education makes self-aware and conscious about one’s health and motivates to engage with the healthcare system at an early stage of illness.

## Intervention Provided to the Depressed Participation With Ptb

All the participants who were found to have depression were provided a psychoeducation session by the interviewer and were referred to the psychiatry out-patient departments of SMIMS and STNMH for management.

## Strength and Limitations

The study included patients with only pulmonary TB unlike other studies previously done where other clinical conditions, such as HIV, were present which may have affected the stigma score. This study also included measures that are specific for patients with TB. Most of the studies conducted on similar variables have not included such measures. However, due to the small sample size, the path analysis should be interpreted as a promising line for further investigation, and its results should be taken with caution. Finally, three of the 30 TBMAS’ items described depressive behaviors (belonging to the three-item factor called mood disorders). The relationship found between TBMAS and depression might have been partially influenced by the existence of these three items. However, the reduced number of items belonging to mood disorders and the fact that we focused on the overall score suggest that our findings can be seen as a promising starting point. Future studies with TBMAS with a larger sample of participants could pay attention to the individual-factor influences on depression and analyze the effects with a larger SEM model with more variables involved.

## Implication of the Study and Conclusion

This study has found an important relationship between different psychosocial variables which can affect medication adherence. We found that internal and perceived stigma affects patients with TB, more among those on the MDR-TB regimen. Many of them develop depression which ultimately affects their adherence to medication. There is an urgent need to integrate mental health with various TB control programs and to include sensitization course in formal education. Patients who are currently suffering from DS-TB may convert to MDR-TB if this cycle of internalized stigma, depression, and poor adherence is not taken care of. Countries, such as India, which is having an increasing number of MDR-TB cases, need to integrate mental healthcare with the National TB control program to reap the maximum benefit.

## Data Availability Statement

The raw data supporting the conclusions of this article will be made available by the authors, without undue reservation.

## Ethics Statement

The studies involving human participants were reviewed and approved by the Sikkim Manipal Institute of Medical Sciences, Sikkim Manipal University. The patients/participants provided their written informed consent to participate in this study.

## Author Contributions

AP, PK, SD, HS, and IS: conceptualization. SB and PG-G: data analysis and interpretation. AP, PK, and SB: manuscript initial drafting. SD, HS, PG-G, and IS: critical analysis. All the authors have read and agreed the final draft and contributed to the article and approved the submitted version.

## Conflict of Interest

The authors declare that the research was conducted in the absence of any commercial or financial relationships that could be construed as a potential conflict of interest.

## Publisher’s Note

All claims expressed in this article are solely those of the authors and do not necessarily represent those of their affiliated organizations, or those of the publisher, the editors and the reviewers. Any product that may be evaluated in this article, or claim that may be made by its manufacturer, is not guaranteed or endorsed by the publisher.
